# Loss of Expression and Function of SOCS3 Is an Early Event in HNSCC: Altered Subcellular Localization as a Possible Mechanism Involved in Proliferation, Migration and Invasion

**DOI:** 10.1371/journal.pone.0045197

**Published:** 2012-09-18

**Authors:** Carlos Rossa, Gunhild Sommer, Luis C. Spolidorio, Steven A. Rosenzweig, Dennis K. Watson, Keith L. Kirkwood

**Affiliations:** 1 Department of Diagnosis and Surgery-School of Dentistry at Araraquara-Universidade Estadual Paulista (UNESP), Araraquara, São Paulo, Brazil; 2 Department of Biochemistry and Molecular Biology, Medical University of South Carolina (MUSC), Charleston, South Carolina, United States of America; 3 Department of Physiology and Pathology-School of Dentistry at Araraquara-Universidade Estadual Paulista (UNESP), Araraquara, São Paulo, Brazil; 4 Department of Pharmacology, Medical University of South Carolina (MUSC), Charleston, South Carolina, United States of America; 5 Department of Pathology and Laboratory Medicine, Medical University of South Carolina (MUSC), Charleston, South Carolina, United States of America; 6 Department of Craniofacial Biology and Center for Oral Health, Medical University of South Carolina (MUSC), Charleston, South Carolina, United States of America; Sun Yat-sen University Medical School, China

## Abstract

**Background:**

Suppressor of cytokine signaling 3 (SOCS3) is an inducible endogenous negative regulator of signal transduction and activator of transcription 3 (STAT3). Epigenetic silencing of SOCS3 has been shown in head and neck squamous cell carcinoma (HNSCC), which is associated with increased activation of STAT3. There is scarce information on the functional role of the reduction of SOCS3 expression and no information on altered subcellular localization of SOCS3 in HNSCC.

**Methodology/Principal Findings:**

We assessed endogenous SOCS3 expression in different HNSCC cell lines by RT-qPCR and western blot. Immunofluorescence and western blot were used to study the subcellular localization of endogenous SOCS3 induced by IL-6. Overexpression of SOCS3 by CMV-driven plasmids and siRNA-mediated inhibition of endogenous SOCS3 were used to verify the role of SOCS3 on tumor cell proliferation, viability, invasion and migration in vitro. In vivo relevance of SOCS3 expression in HNSCC was studied by quantitative immunohistochemistry of commercially-available tissue microarrays. Endogenous expression of SOCS3 was heterogeneous in four HNSCC cell lines and surprisingly preserved in most of these cell lines. Subcellular localization of endogenous SOCS3 in the HNSCC cell lines was predominantly nuclear as opposed to cytoplasmic in non-neoplasic epithelial cells. Overexpression of SOCS3 produced a relative increase of the protein in the cytoplasmic compartment and significantly inhibited proliferation, migration and invasion, whereas inhibition of endogenous nuclear SOCS3 did not affect these events. Analysis of tissue microarrays indicated that loss of SOCS3 is an early event in HNSCC and was correlated with tumor size and histological grade of dysplasia, but a considerable proportion of cases presented detectable expression of SOCS3.

**Conclusion:**

Our data support a role for SOCS3 as a tumor suppressor gene in HNSCC with relevance on proliferation and invasion processes and suggests that abnormal subcellular localization impairs SOCS3 function in HNSCC cells.

## Introduction

The SOCS family of structurally related proteins is mainly characterized as endogenous negative regulators of JAK-STAT signaling. SOCS proteins are induced by cytokines and other stimuli (e.g., insulin, bacterial LPS) and function as negative feedback inhibitors of cytokine signaling. Currently, there are eight members of the so-called CIS-SOCS family described (CIS or cytokine-inducible SH2 protein, and SOCS1-SOCS7), with the best characterized and studied being SOCS1, SOCS2 and SOCS3. These proteins have a similar structural organization that includes: an N-terminal 12 amino-acid domain called kinase inhibitory region (KIR), which is essential for the inhibition of JAK2 kinase [1], [2]; a central SH2 domain responsible for the binding to phosphotyrosine residues in various target proteins and also for the stabilization of SOCS3 [3], [4], [5]; and a C-terminal 40 amino-acid domain called the SOCS box that is responsible for assembly of a protein complex that forms a functional E3 ubiquitin ligase and targets its binding partner for ubiquitin-mediated degradation [6].

**Figure 1:**
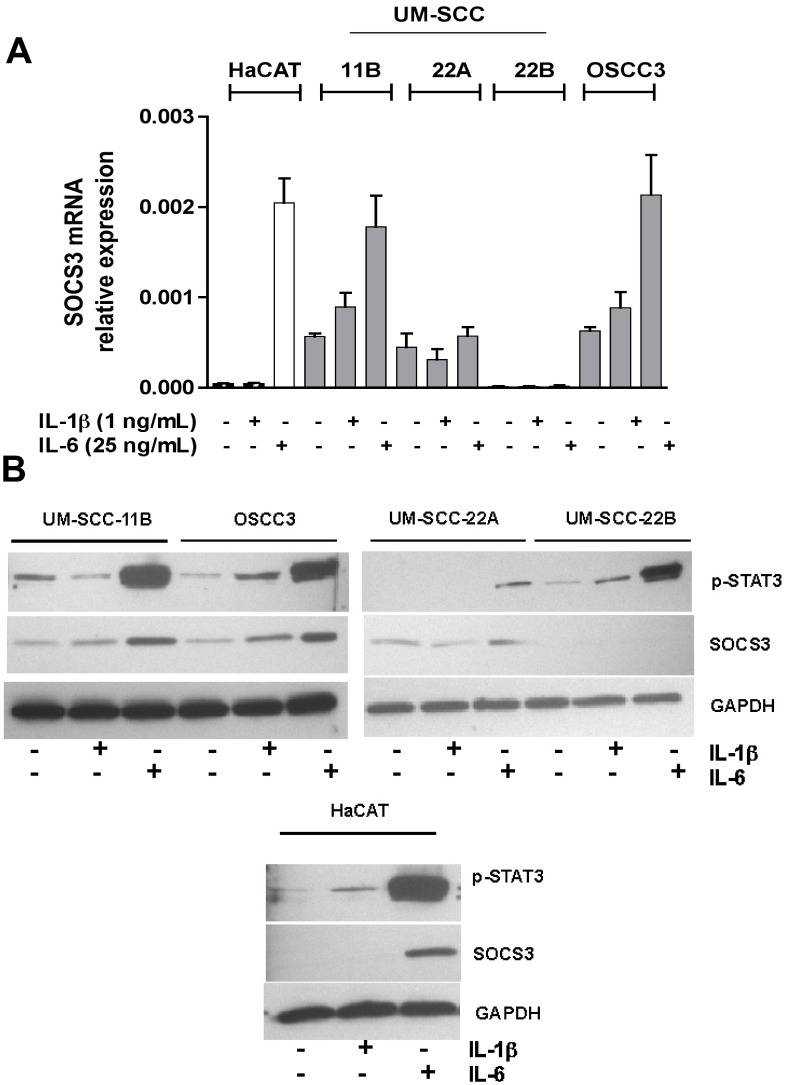
Constitutive and cytokine-induced SOCS3 expression in HSNCC cell lines. OSCC3, UM-SCC-11B, UM-SCC-22A and UM-SCC-22B and one non-neoplastic human epithelial cell line (HaCAT) were evaluated for expression of SOCS3 upon IL-1β and IL-6 stimulation for 18 hours. (A) RT-qPCR results of SOCS3 mRNA normalized to GAPDH. Bars represent mean and vertical lines the standard deviation of 3 independent experiments. (B) Immunoblot results for SOCS3 protein expression and STAT3 activation after stimulation with IL-1 or IL-6. Images are representative of three independent experiments.

**Figure 2:**
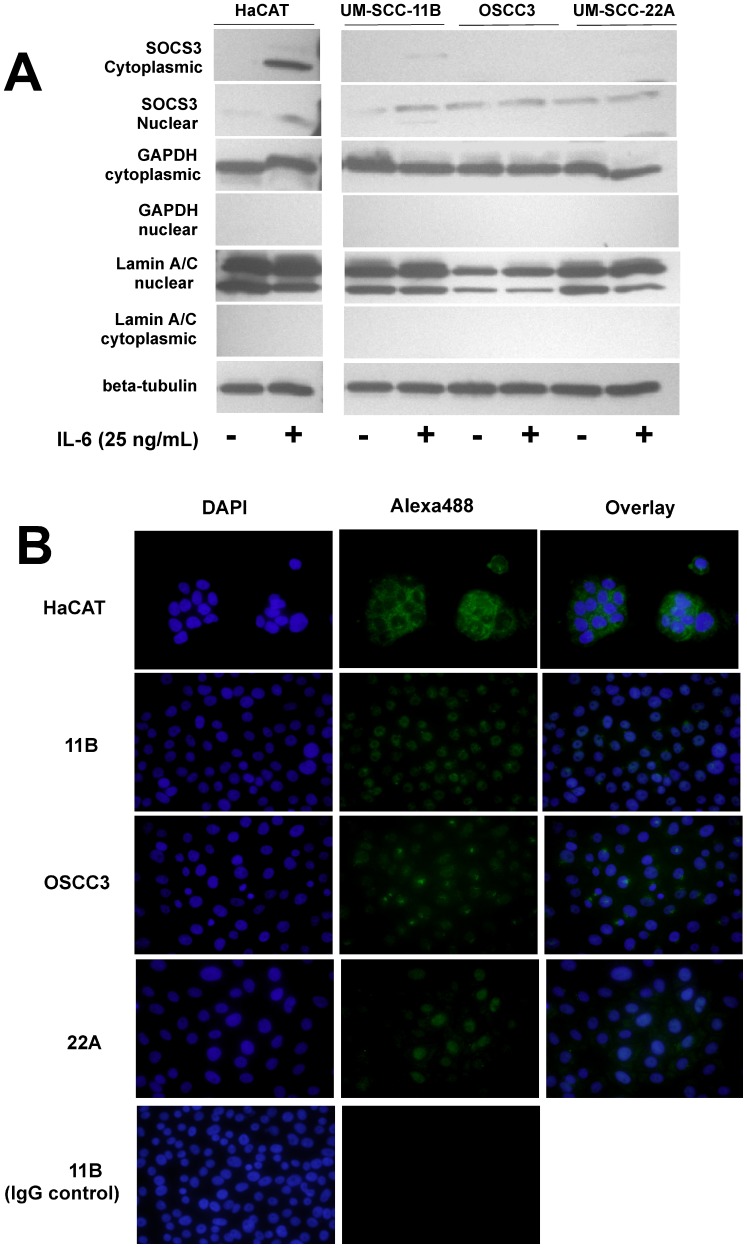
Subcellular distribution of constitutive and IL-6-induced SOCS3 in four different HNSCC cell lines and a non-neoplastic epithelial cell line (HaCAT). (A) Immunoblot of nuclear and cytoplasmic fractions of protein isolated from three HNSCC cell lines and a non-neoplastic epithelial cell line (HaCAT). HNSCC cell lines that still presented some level of SOCS3 expression and the non-neoplastic HaCAT cells were stimulated with IL-6 (25 ng/mL) for 18 hours. These results support the immunofluorescence findings of a nuclear localization of SOCS3 in HNSCC cells, in contrast to the predominantly cytoplasmic localization in HaCAT cells. Expression of nuclear Lamin A/C and cytoplasmic GAPDH were used as controls for the purity of the different protein fractions, whereas beta-actin was used as a loading control for a mixture of equal concentrations of nuclear and cytoplasmic proteins. (B) The indicated cell lines were plated on chamber slides and treated with IL-6 (25 ng/mL) for 18 h to induce SOCS3 expression. After fixation and permeabilization, cells were stained with a rabbit polyclonal antibody against SOCS3 followed with Alexa488-conjugated anti-rabbit IgG secondary antibody. The nuclei of the cells were counterstained with DAPI. Images from random fields were obtained at 600×magnification and are representative of three independent experiments.

Epigenetic silencing of SOCS3 has been reported in head and neck squamous cell carcinoma (HNSCC) [7], suggesting that decreased expression of SOCS3 could represent an important cause of constitutive JAK/STAT activation in HNSCC and supporting the notion that SOCS3 could function as a tumor suppressor gene. This notion is further supported by the finding that restoring SOCS3 expression in tumor cell lines results in growth suppression and induction of apoptosis [7]. However, there is significant heterogeneity of SOCS gene expression in various types of cancer, including HNSCC, and there is no information on the relevance of the loss of SOCS3 for HNSCC tumor progression or correlation with tumor size and grade of dysplasia. Increased expression of SOCS3 is associated with cutaneous T-cell lymphoma, some acute leukemias and hepatocellular carcinoma [8], [9], [10], [11]. In these examples, expression of SOCS3 may be a natural consequence of increased STAT3 activation and cytokine production by tumor cells. In these cancer cells, different mechanisms may account for sustained STAT3 activation, including the failure of other negative regulatory pathways of JAK-STAT signaling which would overwhelm the capacity of SOCS proteins to dampen STAT activation [12].

SOCS3 has been reported to bind to cytokine receptor chains with high affinity, especially gp130 receptors. This mechanism and the proteasome-mediated degradation of SOCS3 binding partners presuppose its expression in the cytoplasm for adequate function [3], [13]. In the present study we show that altered subcellular localization is an additional mechanism of SOCS3 loss of function in oral cancer cells. Similarly to the already shown epigenetic silencing of SOCS3, changes in its subcellular localization affect cell proliferation and invasion and this mechanism may be occurring in the cases that still present detectable SOCS3 expression. We present evidence that overexpression of SOCS3 in HNSCC cell lines that express or do not express endogenous SOCS3 has different impacts on *in vitro* proliferation and invasion in different HNSCC cell lines and confirm the relevance of subcellular localization of SOCS3 by siRNA experiments in an oral cancer cell line that retains detectable endogenous expression of SOCS3. Finally, we also report that decreased SOCS3 expression is an early event in HNSCC, suggesting that SOCS3 may constitute an interesting therapeutic target for these tumors.

**Figure 3:**
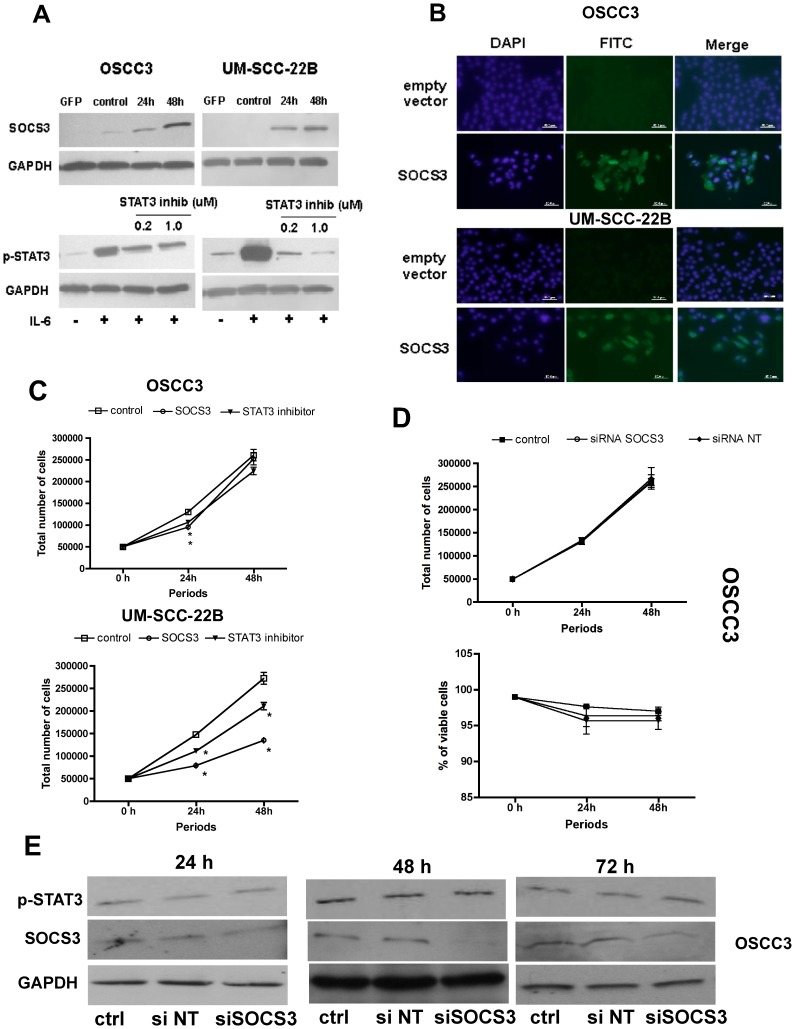
Modulation of SOCS3 expression in gain and loss of function experiments in HNSCC only reduces proliferation of cells without (UM-SCC-22B) detectable levels of endogenous SOCS3. (A) Immunoblot analysis of whole cell extracts shows that transfection of the SOCS3 expression plasmid resulted in increased levels of SOCS3 after 24 and 48 h in OSCC3 and UM-SCC-22B cells. Pre-treatment of both cell lines with the indicated concentrations of the biochemical inhibitor effectively decreased phosphorylation of STAT3 30 min after stimulation with IL-6 (25 ng/mL). (B) Subcellular localization of SOCS3 in the overexpression experiments was confirmed by immunofluorescence analysis. Images are representative of three independent experiments assessing SOCS3 transgene expression 48 h after plasmid transfection. (C) Cell proliferation was determined by direct counting the cells and the trypan blue dye exclusion test 24 and 48 h after transfection. (D) Cell proliferation was also determined in loss of function experiments by direct counting of cells using trypan blue exclusion test 24 and 48 h after siRNA transfection in OSCC3 cells. Graphs (C and D) represent growth curves (number of viable cells) over time, according to the experimental condition (in C: transfection of empty vector, SOCS3 expressing vector, or treatment with STAT3 inhibitor; in D: reagent control, SOCS3 siRNA and non-target control siRNA). Vertical lines standard deviations of three independent experiments and * indicates p<0.05 for comparison with vehicle control (empty vector or non-targeting siRNA in gain and loss of function experiments, respectively). (E) Immunoblot analysis verifying the efficiency of siRNA-mediated inhibition of endogenous SOCS3 expression 24, 48 and 72 h after transfection. Images are representative of three to five independent experiments.

## Materials and Methods

### Cell Lines and Reagents

HNSCC cells from the University of Michigan collection (UM-SCC-11B, 22A and 22B) were a kind gift from Dr. Thomas E. Carey (University of Michigan, Ann Arbor, MI). OSCC3 and HaCAT cells were gifts from Drs. Jacques E. Nor and Nisha D’Silva (University of Michigan, Ann Arbor, MI), respectively. All cells were grown in DMEM supplemented with 10% heat-inactivated fetal bovine serum, 100 U/mL penicillin and 100 µg/mL streptomycin (all from Invitrogen Corp., Carlsbad, CA). SOCS3 primary antibodies were from Abcam (Cambridge, MA) and Cell Signaling (Beverly, MA), phosphorylated STAT3, lamin A/C, GAPDH and beta-tubulin antibodies were all from Cell Signaling. STAT3 biochemical inhibitor was purchased from Roche (San Francisco, CA). SOCS3 expression plasmids and the empty control vector and GFP-expressing plasmid were obtained from Origene (Origene Technologies Inc., Rockville, MD). SOCS3 pool siRNA was from Dharmacon (Thermo Fisher Scientific Inc., Lafayette, CO). Recombinant cytokines were obtained from R&D systems (Minneapolis, MN). Matrigel invasion chambers, secondary fluorescent-conjugated and IgG control antibodies were purchased from BD Biosciences (Bedford, MA). Tissue microarrays were purchased from Imgenex (Imgenex Corp., San Diego, CA), Accumax (ISU ABXIS Co, Korea) and Biomax (US Biomax Inc., Rockville, MD). All reagents for immunohistochemistry were from Dako (Glostrup, Denmark).

**Figure 4:**
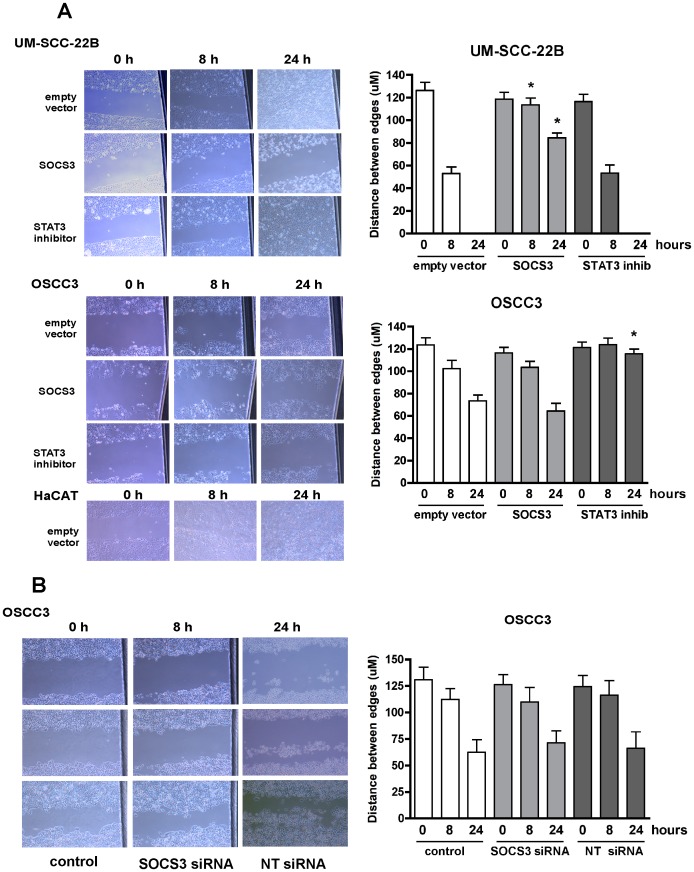
Differential effects of modulation of SOCS3 expression and STAT3 inhibition on HNSCC migration. (A) Representative phase-contrast images of the in vitro wound area in the different cell cultures (100×magnification) for the gain of function experiments by transfection of a CMV-driven SOCS3 expression plasmid or the empty vector control. STAT3 biochemical inhibitor was used to assess possible effects of SOCS3 that are independent of modulation of STAT3 activity. The distance between the edges of the wound was measured on digital images captured adjacent to the reference notch made in the cell culture plastic (black area on the right of the images) Non-neoplastic HaCAT cells were transfected with the empty vector to control for non-specific effects of the transfection procedure on cell migration. Bars represent averages and vertical lines standard deviation of three independent wounding experiments, measured in triplicate (*p<0.05 indicates significant difference in comparison to the distance between edges of the wound at the same time period in empty-vector transfected cells). (B) Representative phase-contrast images of the in vitro wound area in the loss of function experiments by transfection of SOCS3 siRNA or non-targeting siRNA in OSCC3 cells. Quantitation of cell migration was performed as described in (A) and the graph represent average and standard deviations of three independent wounding experiments measured in triplicate.

**Figure 5:**
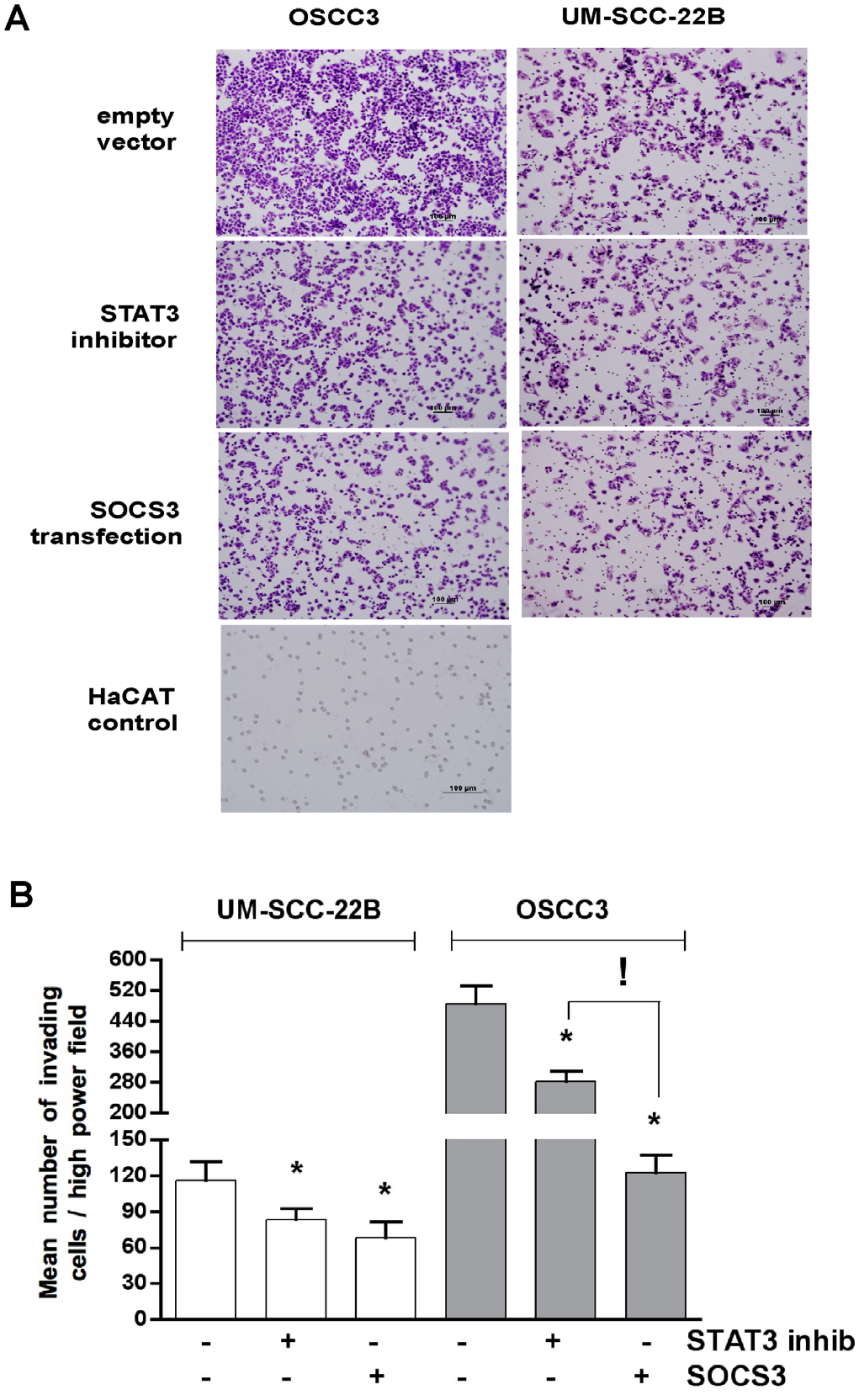
Gain of function of SOCS3 reduces HNSCC invasion in vitro. (A) Representative images of lower aspect of the transwell membrane after removal of the matrigel on its upper aspect and staining of the cells with the Diff-Quick kit (200×magnification). The non-neoplastic epithelial cell line (HaCAT) was used as a control and did not invade the matrigel layer. (B) Invading cells in the lower part of the transwell were counted in 6 random high-power fields (400X) by an examiner that was blind to the experiment purposes. Three independent experiments were performed and the bars indicate averages and vertical lines the standard deviations (*p<0.05 in comparison to empty vector-transfected cells, *p<0.05 between indicated groups).

### RT-qPCR

Complementary DNA was be synthesized by reverse transcription of 500 ng of total RNA using 2.5 µM Oligo (dT) 16 primers and 1.25 U/µL Moloney murine leukemia virus reverse transcriptase in the presence of 5.5 mM MgCl_2_, 2 mM dNTPs and 0.4 U/µL of RNAse inhibitor, according to the manufacturer’s protocol (Applied Biosystems). 2 µL of the RT reaction product were used on a 20 µL total volume qPCR reaction mix that included nuclease-free water, TaqMan universal PCR master mix and TaqMan gene expression assays including primers and probe (Applied Biosystems, assay ID# Hs02330328_s1) for detection of human SOCS3. The expression of target genes was normalized for the levels of house-keeping GAPDH and relative quantities were determined by the relative Ct method with the thermocycler’s software (StepOne Plus, Applied Biosystems).

**Figure 6:**
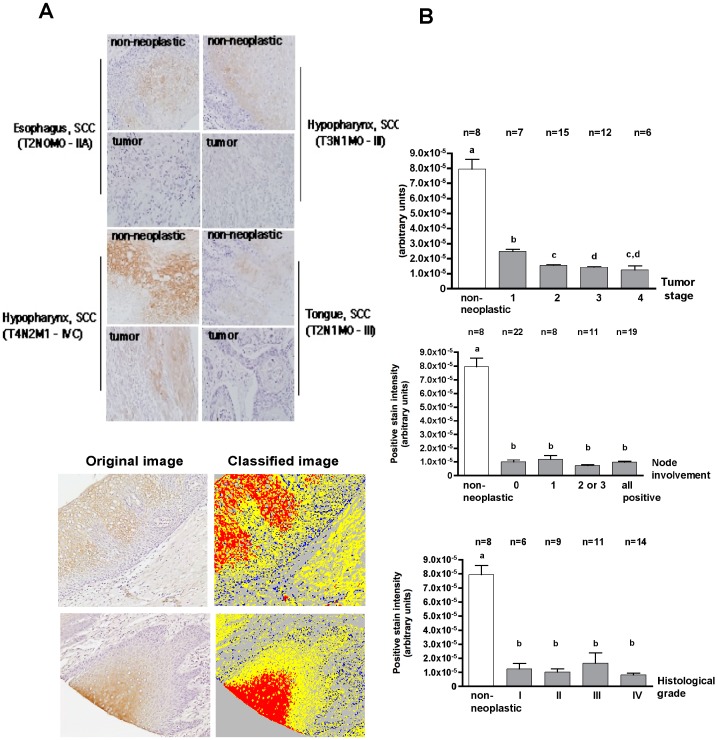
Decrease of SOCS3 expression is an early event in head and neck cancer. Immunohistochemical staining for SOCS3 in four different tissue microarrays was analyzed with a digital slide scanner system. The intensity of positive staining was normalized to the area of positive staining to account for the larger area occupied by epithelial cells in the tumor samples. (A) Representative images of the results for immunohistochemical staining of SOCS3 are shown for 4 of the squamous cell carcinoma (SCC) cases that had both neoplastic and non-neoplastic samples with the indicated pathological information (location, TNM staging) (400X). Below are representative images of the classification system with the slide scanner. The protocol designed recognizes positive staining (in red), non-stained tissue (in yellow), cell nuclei (in blue) and empty spaces (in gray) (100X). (B) Results for the normalized intensity of SOCS3 expression according to tumor staging classification. The number of cases analyzed in each condition is indicated above the graphs (‘n’). This number varies because some TMAs did not have all the staging information and because some spots were lost during the staining process. Bars represent averages and vertical lines the standard deviations. Different letters above the columns indicate a statistically significant difference (p<0.05).

### Overexpression of SOCS3

Transfection of CMV-driven SOCS3 plasmid constructs, empty vector control and GFP-expressing plasmid was performed with the Nucleofection system. Briefly, 1×10^6^ cells were nucleofected using 2 µg of plasmid DNA using program U-031 and the V nucleofection solution, according to the instructions of the manufacturer (Amaxa, Lonza, Basel, Switzerland). Minimum transfection efficiency was approximately 40%, reaching up to 60% in the different cell lines (data not shown).

### Silencing of SOCS3

Transfection of ON-TARGET*plus* SMARTpool siRNA for human SOCS3 or the ON-TARGET*plus* non-targeting pool siRNA (Dharmacon, Thermo Fisher Scientific) was performed with the Neon transfection system. Briefly, 1×10^6^ cells were transfected using 100 nM siRNA in 100 µL volume using 1,450 V and 10 ms, conditions optimized according to the instructions of the manufacturer (Invitrogen, Life Technologies). Efficiency of silencing of the target gene was assessed by western blot 48 and 72 h after transfection. Cells were always plated 24 h after transfection and subsequent experiments initiated after an additional 24 h (i.e., 48 h after transfection of siRNA).

### Western Blot

Protein was obtained by preparation of cell lysates in M-Per (Pierce) supplemented with protease and phosphatase inhibitors (Roche). For the experiments evaluating cytosolic and nuclear fractions, we used NE-PER nuclear protein extraction kit (Pierce), according to the supplier’s instructions. These were quantitated using a commercial Lowry microtiter assay (Bio-Rad DC assay), and 30 µg of total protein (or 20 µg of nuclear and cytoplasmic fractions) were be mixed with SDS sample buffer, followed by heat-denaturation. Electrophoretic separation was conducted on 10% Tris-Cl polyacrylamide gels and subsequently electro-transferred to nitrocellulose membranes. These membranes were incubated with primary antibodies for SOCS3 and p-STAT3 overnight at 4°C and the presence of the primary antibodies was detected with HRP-conjugated secondary antibodies using a chemiluminescence system (LumiGlo, Cell Signaling). As loading controls we used primary antibodies for GAPDH and beta-tubulin, whereas the separation of cytosolic and nuclear fractions was confirmed by the detection of GAPDH and lamin A/C.

### Invasion and Migration Assays

Migration was assessed by the scratch assay, as described previously [14]. Cells were plated in 6-well plates in densities that allowed a confluent layer in 24 h. Standardized wounds were prepared by scratching the monolayer of cells with a P200 pipet tip. Two reference notches were made on the plastic at the bottom of the wells with a sterile scalpel blade to allow standardization of the microscopic filed monitored over time. Digital images were then obtained from the same microscopic field after 8 and 24 h on a inverted microscope (Nikon Eclipse, Nikon, USA) and the distance between the edges of the wound measured with an imaging software (NIS Elements, Nikon, USA).

We used a commercially-available assay (BD Biocoat Matrigel Invasion Chambers) according to the supplier’s instructions (BD Biosciences). Briefly, Matrigel inserts were rehydrated in culture medium and subsequently transferred using sterile forceps to another well containing 0.75 mL of medium supplemented with a chemoattractant (5% FBS). 0.5 mL of a 5×10^4^ cell suspension were added to the top of the chamber and incubated in a humidified atmosphere at 37 C and 5% CO_2_ for 22 hours. Non-invading cells were removed by two consecutive gentle scrubs with moist sterile cotton swabs and the inserts stained with Diff-Quick staining kit. The inserts were air-dried and the matrigel membrane mounted face-down in microscope slides. The number of cells was counted in six microscopic fields of triplicate membranes by a single observer blind to the experimental purpose and averaged.

### Immunohistochemistry and Immunofluorescence

For immunofluorescence detection of SOCS3, cells were plated in chamber slides, and after experimental treatment fixed with paraformaldehyde, permeabilized with saponin (BD Cytofix/Cytoperm, BD Biosciences) and the slides were incubated with primary antibodies against SOCS3 for 1 h or with the same concentration of irrelevant rabbit IgG. After washing, the slides were incubated for 30 min with goat anti-rabbit secondary antibodies conjugated to AlexaFluor 488 at 4 C and protected from light. Digital images from three random fields were obtained at 600×magnification on a fluorescent microscope (Olympus BX61, Olympus America, Philadelphia, USA) with image analysis software (FluoImager, Visiopharm, Hoersholm, Denmark).

Immunohistochemistry detection of SOCS3 was initially optimized in slides of various human tissues (lung, skin, liver) and in test tissue microarrays (data not shown). Staining of the tissue microarrays was performed using the optimized conditions, including dilution of primary SOCS3 antibody and incubation time, according to the instructions of the suppliers of the TMAs. Briefly, the external coat of paraffin was removed by baking the slides at 60 C for 2 h, followed by complete de-paraffinization in xylene and rehydration in graded series of ethanol. Primary anti-SOCS3 antibody or irrelevant IgG control from the same species were incubated with the slides and detected with a biotin-streptavidin/DAB system (LSAB2, Dako USA) and counter-stained with hematoxylin. The TMAs were then scanned on a digital slide scanner (Olympus BLISS system, including BX61 Research Microscope with motorized stage and DP72 digital camera, Olympus America, Philadelphia, USA) and the image analysis software (Visiopharm ArrayImager and Image Analysis, Visiophorm, Hoersholm, Denmark) was calibrated to detect positive (shades of brown), non-stained tissue (blue, light blue, shades of gray) and empty clear areas without any tissue. Readout was the intensity positive staining, which was normalized to the area of positive staining.

### Statistics

Comparison between experimental conditions was performed by non-paired t-test using GraphPad Prism version 4.00 for Mac (GraphPad Software, San Diego California USA, www.graphpad.com), assuming independence between the two conditions compared. Significance levels was always set at 95%.

## Results

### Head and Neck Squamous Cell Carcinoma Cell Lines Express Various Levels of Endogenous SOCS3 mRNA and Protein, but All Cell Lines are JAK-STAT Responsive

Initially, we screened four HNSCC cell lines for expression of SOCS3 at the mRNA and protein level and found significant heterogeneity. Three HNSCC cell lines (OSCC3, UM-SCC11B and UM-SCC-22B) also showed some level of constitutive activation of STAT3 (Figure 1). The 18 h stimulation period was selected to allow assessment of steady-state mRNA levels. Endogenous SOCS3 expression was preserved in three out of the four HNSCC cell lines, which interestingly did not require induction by activation of gp130/STAT3 signaling. In two cell lines (OSCC3 and UM-SCC-11B) significant induction of SOCS3 mRNA was observed in response to IL-6 stimulation, which was also observed in the non-neoplastic cell line (HaCAT) indicating the responsiveness of JAK-STAT signaling. At this 18 h period, transcription-translation coupling of SOCS3 was preserved in all cell lines. Two cell lines (OSCC3 and UM-SCC-11B) retained expression of SOCS3 at mRNA and protein level, which was significantly inducible by IL-6, whereas two other cell lines had low (UM-SCC-22A) or negligible (UM-SCC-22B) expression of SOCS3. All HNSCC cell lines were JAK-STAT responsive, and three of the four cell lines exhibited constitutive activation of STAT3.

### Nuclear Localization of SOCS3 in Three Out of Four HNSCC Cell Lines Used

Since in OSCC3 and UM-SCC-11B cell lines SOCS3 expression was retained, we evaluated if the negative regulation of signaling mediated by SOCS3 was affected by its localization in the cells. SOCS3 functions in the cytoplasm, where it can exert its inhibitory roles by binding to the cytoplasmic portion of transmembrane receptors and inhibit phosphorylation events through its SH2 and KIR domains, or by binding to various signaling intermediate kinases through the SOCS box and ultimately targeting them to degradation by the proteasome. Indeed, we observed that SOCS3 expression was primarily located in the nucleus of three out of four HNSCC cell lines used in this study, as opposed to the normal epithelial cell line (HaCAT), in which SOCS3 was mainly located in the cytoplasm. These results were confirmed by immunoblotting of nuclear and cytoplasmic fractions and immunofluorescence analysis on fixed and permeabilized cells (Figure 2, A and B) of the three HNSCC cell lines that retained expression of SOCS3.

### Gain of Function of SOCS3 Reduces HNSCC Proliferation, Migration and Invasion of in vitro. Loss of Function by Silencing Endogenous SOCS3 that is Localized Mostly in the Nucleus has no Effect on HNSCC Proliferation and Migration

We chose OSCC3 and UM-SCC-22B cell lines for gain of function experiments by overexpression because these are representative of cells with endogenous SOCS3 expression and cells in which SOCS3 is not detectable, respectively. Because OSCC3 cell line retained endogenous expression of SOCS3, which was mostly nuclear, this cell line were also used in the siRNA, loss-of-function experiments. In the conditions used in these experiments, both cell lines showed greater than 40% transfection efficiency, as evaluated by the transfection of a control GFP expression plasmid of similar size (data not shown), whereas the siRNA efficacy in OSCC3 cells (that retained endogenous expression of SOCS3) was monitored by western blot 24, 48 and 72 h after transfection. Interestingly, the levels of phosphorylated STAT3 were not affected by loss of function of SOCS3, suggesting that its predominantly nuclear localization had no functional role in regulating STAT3 activation in this cell line (Figure 3, E). To obtain insight into effects of SOCS3 that may not be mediated through inhibition of STAT3, we also used a biochemical inhibitor of STAT3. Modulation of SOCS3 expression in HNSCC cell lines was maximum 48 h after transfection in both cell lines and for gain of function (overexpression by CMV-driven plasmid - Figure 3, A and B) and loss of function (siRNA, Figure 3, E), so this period was used for all the subsequent experiments involving gain and loss of function. Interestingly, in the gain of function experiments 48 h after plasmid transfection, SOCS3 transgene expression was located mostly in the cytoplasm and thus, would be expected to function properly (Figure 3, B). Cell proliferation was significantly decreased following SOCS3 overexpression and treatment with the STAT3 inhibitor in both HNSCC cell lines at 24 h and only in the UM-SCC-22B, which did not express endogenous SOCS3, at 48 h (Figure 3, C). The loss of function experiments using siRNA in OSCC3 cells did not result in significant changes on cell proliferation or viability, indicating that the endogenous expression of SOCS3 in an altered subcellular localization was indeed not relevant for cell proliferation (Figure 3, D).

To evaluate whether HSNCC invasion would be affected by gain of function of SOCS3, we initially assessed cell migration using a ‘scratch assay’ (Figure 4, A). Surprisingly, in the UM-SCC-22B cells that do not express detectable endogenous SOCS3, we observed a significant inhibition of *in vitro* migration with SOCS3 overexpression, which was an effect that was not mediated by inhibition of STAT3. On the other hand, in OSCC3 cells inhibition of STAT3 resulted in a small but significant inhibition of migration, whereas SOCS3 overexpression did not (Figure 4, A). Loss of function experiments by siRNA-mediated inhibition of endogenous SOCS3 expression in OSCC3 cells did not change *in vitro* migration of this cell line, suggesting that SOCS3 does not play an important role in migration for this cell line (Figure 4, B). *In vitro* invasion was significantly inhibited in both HNSCC cell lines by overexpression of SOCS3 and by the STAT3 inhibitor. However, SOCS3 overexpression resulted in a significantly more potent effect than the STAT3 inhibitor in OSCC3 cells (Figure 5).

### Decrease of SOCS3 Expression is an Early Event in HNSCC

Considering the role of SOCS3 in HNSCC cell proliferation, migration and invasion *in vitro* and the fact that some cancer cell lines retained endogenous expression of SOCS3, we next wanted to assess its expression in HNSCC according to the size and grade of the tumors. We used four commercially-available tissue microarrays obtained from three different companies to evaluate SOCS3 expression by immunohistochemistry in various head and neck cancer cases classified by the TNM system as stage 1 through 4 in size, dysplasia grade I through IV, with and without nodal involvement. Digital images of these TMAs were obtained and analyzed with a slide scanner system, which objectively quantified the area and intensity of positive (brown) staining (Figure 6-A). The data derived from the slide scanner was validated by an experienced oral pathologist who independently scored the tissue microarrays for SOCS3 expression using the H-score method (data not shown). This pathologist did not have access to the pathological information available the tissue arrayed. Overall the results indicate a significant reduction in SOCS3 expression in head and neck cancer in comparison to non-neoplastic tissues, and this decrease was observed at early stages (Figure 6-B), and even though we could not assess the subcellular localization of SOCS3 by immunohistochemistry some cases retained detectable expression levels. Overall, this data suggest that loss of SOCS3-mediated regulation of signaling is involved in oncogenesis.

## Discussion

The role of SOCS3 in various types of cancer is controversial. There are reports associating either increased or reduced expression of SOCS3 with breast [15], [16] and prostate cancer [17], [18]. Our data support a role for SOCS3 as a tumor suppressor gene in HNSCC, which agrees with its proposed role in melanoma [19], lung [20] and liver cancer [21]. Most studies associate the silencing of SOCS3 expression by epigenetic mechanisms with the occurrence and progression of these tumors and HNSCC [7], [19], [20], [21]. Our data indicate that the loss of SOCS3 expression in HNSCC is an early event that is maintained and even discretely accentuated with tumor progression, implicating this gene not only in tumorigenesis but also in tumor progression and invasion. However, our data also shows that SOCS3 expression is not completely abrogated in HNSCC and our *in vitro* data with HNSCC cell lines also demonstrates an heterogeneous expression of SOCS3, as three out of four cell lines presented endogenous expression at mRNA and protein levels. Overall, this result is similar to recent data indicating an inverse relationship between SOCS3 expression and tumor stage and clinical outcome of breast cancer [15]. Loss of SOCS3 expression has also been recently associated with increased risk of recurrence of breast cancer [22]. The relationship between SOCS3 expression and OSCC tumor invasion is further supported by our *in vitro* data, which suggests that overexpression of SOCS3 decreases HNSCC invasion by different mechanisms, affecting primarily migration in cells that do not express detectable endogenous SOCS3 (UM-SCC-22B) and in cells that retain detectable endogenous expression of SOCS3 it may also modulate expression of genes that are important for invasion. Loss of function experiment using siRNA in a cell line presenting endogenous SOCS3 did not affect proliferation or migration, suggesting that the SOCS3 expressed in the nucleus of this cell line may not be functional. Thus, preventing the synthesis of SOCS3 protein had no effect. The finding that SOCS3 overexpression can affect HNSCC invasion *in vitro* by different mechanisms is important and indicates that this can be a potential therapeutic target.

HNSCC are acknowledged as highly heterogeneous tumors, and we verified that expression of SOCS3 is also variable in different HNSCC cell lines. This could suggest that the potential relevance of the role played by SOCS3 in HNSCC may vary in different cases. Previous findings indicate epigenetic silencing of SOCS3 as a major mechanism for the loss of function of SOCS3 [7], but the fact that cell lines and some HNSCC cases still present SOCS3 expression and have similar invasive biological behavior is intriguing and suggest an alternative mechanism for the impaired function of SOCS3, if this gene is relevant for tumor progression. Interestingly, some HNSCC cells even exhibited constitutive expression of SOCS3 mRNA and protein, which was not observed in normal epithelial cells.

One mechanism for the lack of functional activity of SOCS3 in HNSCC cells that we describe for the first time in HNSCC cells is altered subcellular localization. A clear contrast in SOCS3 localization was observed in non-neoplastic epithelial cells compared to HNSCC cell lines. Because SOCS3-mediated suppression of gp130 receptor signaling is via association with the cytoplasmic portion of the receptor [13], a predominant nuclear localization would be expected to impair its function. This is supported by reduced proliferation, migration and invasion induced by overexpression of SOCS3 in HNSCC cell lines that lacked endogenous expression of this protein (UM-SCC-22B). Moreover, siRNA-mediated inhibition of endogenous SOCS3 localized predominantly in the nucleus of OSCC3 cells did not affect proliferation or migration, indicating that this endogenous SOCS3 has no functional activity. Interestingly, the SOCS3 promoter in this cell line was functional and responsive, since mRNA expression was induced by cytokine stimulation. A nuclear localization signal has been identified and characterized for SOCS1 but not for SOCS3 [23]. The subcellular localization of SOCS3 may be dependent on its interaction with other proteins, such as the small microtubule-associated protein 1 (MAP1S), known to bind the SOCS3 C-terminal domain and function as a cytoplasmic anchor [24]. Evidence exists for an antitumor role of altered stability and organization of cytoskeleton and microtubule systems in cancer [25]; microtubule inhibitors (i.e., docetaxel and paclitaxel) have long been used as chemotherapeutics [26]. A possible oncogenic role for microtubule-associated protein 2A (MAP2A) was suggested in oral cancer [27], [28].

Importantly, the physical association of SOCS3 and MAP1S has been shown to be necessary for the functional attenuation of STAT3 activation induced by IL-6 in macrophages [24]. In our overexpression experiments, SOCS3 expression was increased in the nucleus but its cytoplasmic localization was also increased relative to untransfected cells, which may explain the enhanced functional activity of SOCS3 in these experiments. In contrast, it has been suggested that nuclear localization of SOCS3 requires increased cytoplasmic levels [29], consistent with the findings we report in this study that overexpression of SOCS3 increased both nuclear and cytoplasmic localization. Moreover, other evidence indicates that SOCS3 is localized both in the cytoplasm and nucleus in normal and tumor cell lines [23]. This suggests that SOCS3 may have a functional role in the nucleus, similar to that described for nuclear localization of SOCS1, which is required for complete inhibition of interferon (IFN)-α induced gene expression, IFN-γ-mediated CD54 expression [23] and for transcriptional termination of NF-kB signaling leading to its proteasomal degradation [30], [31]. Although a functional role for nuclear SOCS3 has not been shown, it will be interesting to determine if it has a role in HNSCC cells. It is important to bear in mind that some SOCS3 expression was significantly reduced but not completely eliminated in most cases of HNSCC included in the TMAs analyzed, supporting the notion that a loss of function mechanism may be involved in addition to epigenetic silencing.

Our results also indicate that the reduced expression and functional activity of SOCS3 is of relevance for HNSCC cell proliferation, migration and invasion suggesting SOCS3 has a role in HNSCC tumor progression. However, the influence of SOCS3 in these processes may be mediated, in part by a prominent role of STAT3 activation in the tumor cells, as the decrease in cell proliferation and invasion *in vitro* by overexpression of SOCS3 was less marked in a cell line (OSCC3) in which a biochemical STAT3 inhibitor was also less effective; other mechanisms may be compensating for the inhibitory function of SOCS3 and of the biochemical inhibitor on STAT3 activation, such as let-7a microRNA which contributes to constitutive STAT3 phosphorylation in malignant cholangiocytes [32]. STAT3 relevance in oncogenesis/tumor progression is widely acknowledged, via regulating apoptosis-related genes (Bcl-X_L_, Mcl-1 and Survivin) [33], proliferation- (c-myc and cyclin D1) and invasion-promoting genes (VEGF, MMP-9) [34], [35].

While STAT3 may be the primary target, SOCS3 has a broader range of activity and can modulate other signaling pathways such as inhibition of TNF-receptor-associated factor 6 (TRAF6) and transforming growth factor-β (TGFβ)-activated kinase 1 (TAK1) [36], which are crucial for activation of MAP Kinases and NF-kB. Because the role of SOCS3 in HNSCC cells is unknown, future studies are needed to directly examine the effects of SOCS3 on HNSCC cells and its modulation of signaling pathways other than STAT3.

Overall, our results indicate that SOCS3 has a tumor suppressor role in HNSCC, since its expression is reduced early in tumor development; and the overexpression of SOCS3 in HNSCC cell lines reduces proliferation, migration and invasion. Moreover, we propose that the loss of SOCS3 function may be associated not only with reduced expression due to epigenetic silencing but also with abnormal subcellular localization impairing its function. Indeed, in these same cell lines, we observed that DNA methyl-transferase-1 and histone deacetylase inhibitors partially affect SOCS3 expression (data not shown), but we cannot state that this effect is direct or indirect, nor we have studied the status of SOCS3 promoter methylation. However, since future studies will explore the epigenetic silencing of SOCS3, including mapping the sites of methylation and changes on the histone code. As a putative tumor suppressor gene, determination of expression and subcellular localization of SOCS3 may be useful as a diagnostic tool or as a therapeutic target in gene therapy studies.
